# Assessment of Willingness to Pay for Pollution Prevention, Health and Happiness: A Case Study of Punjab, Pakistan

**DOI:** 10.3389/fpubh.2022.825387

**Published:** 2022-06-20

**Authors:** Muhammad Zahid Rafique, Jinping Sun, Abdul Razaque Larik, Yafei Li

**Affiliations:** ^1^Centre for Economic Research, Shandong University, Jinan, China; ^2^General Department, Government College University, Hyderabad, Sindh, Pakistan; ^3^School of Economics, Peking University, Beijing, China

**Keywords:** air pollution, exposure assessment, happiness, tax payment, social contribution

## Abstract

Air pollution has been notoriously held accountable for a substantial number of deaths in several countries. Moreover, its negative impact on people's health and well-being has also been witnessed in countries where air pollution is below the recommended national levels. The urban cities of Pakistan are among the worst South Asian areas in terms of air pollution. Because of this problem, the health and well-being of citizens are affected. The present study investigates the impact of air pollution on urban residents' happiness and health. It analyzes their willingness to pay for pollution prevention and its determinants by employing the data obtained through a primary survey. Pakistanis are unaware of air pollution's effect on health and quality of life, therefore only 12.5% consider this problem very serious. The results confirm the significantly negative effect of air pollution on happiness. Concerning the willingness to pay, it is differentiated in the form of tax and social contribution. Pakistanis are willing to pay more in social contribution in return for different environmental attributes. The results show that only 13% of respondents are not willing to pay for income contribution to improve air quality reporting indifferent attitude and insufficient knowledge of the environment. Our findings suggest that their apprehension concerning the environment influences people's willingness to pay. The study concludes that despite Pakistan's underdeveloped economic stature and its poor and flexible budgetary allocation for the betterment of air quality, most Pakistanis showed their willingness to pay for environmental protection. The government and environmental organizations ought to generate consensus among the general population about environmental importance, individual responsibility, and social duties thereby lessening the free-rider problem and reducing air pollution for better social welfare.

## Introduction

Air pollution has drawn equal attention of researchers from environmental and economic sciences owing to its multifaceted negative impact on health and the economy (1). Air quality affects a person's utility of public good. An individual's willingness to pay (WTP) taxes for the betterment of air quality can serve as the main factor when an exchange between economic development and environmental regulations takes place (2). In this respect, since 1990s, there has been a growing trend toward studying well-being (or “happiness research” or “quality of life research”) theoretically or empirically (3–8). Nevertheless, the life satisfaction valuation approach employed in environmental valuation is considered an unconventional technique that can hardly be found in current standard literature. The life satisfaction method takes into consideration the environmental advantages of decrease in air pollution, which, in turn, can exert a direct impact on individual well-being (experienced utility) in the spheres of physical and psychological health, recreation, and aesthetics (9–12). The majority of countries worldwide have advanced significantly and comprehensively in terms of well-being excepting the natural environment (13). Urban air pollution tops the list of problems emanating from environmental degradation faced by the urban population. Growing empirical evidence categorically suggests that excessive amounts of suspended particulates cause health-related problems creating wide-ranging health hazards particularly affecting lungs and heart. The most harmful among these are the fine particulates of 10 microns or smaller in diameter. PM_2.5_ is a fine air pollution particle that can penetrate deep into the human body. The latest research clearly indicates that exposure to even lower concentrations of PM_2.5_ can raise the chances of critical health issues (14, 15). Pakistan, India, Bangladesh, and China have recorded “increasing trends in PM_2.5_ exposure”. While China is known to have improved dealing with pollution, Pakistan, India and Bangladesh “have experienced the steepest increases in air pollution levels since 2010 and now present the highest sustained PM_2.5_ concentrations” (15).

With an alarming figure of estimated 35% of people residing in urban areas, Pakistan is considered the largest urbanized state in the South Asian region (16). Like elsewhere in the developing economies, the urban cities in Pakistan keep on expanding in size and population, providing versatile, unprecedented employment opportunities, convenience, and facilities that are largely unavailable in other parts of the country. However, all that has come at the costs of environmental degradation in the shape of increased amount of pollution, garbage, congestion and the damage to ecosystem. The Pakistani urban area statistics suggest that the PM concentration in urban Pakistan is far greater in comparison to its Bhutanese and Sri Lankan counterparts in the South Asian region. Pakistan has been known to be incapable for systematic monitoring of PM_2.5_. Furthermore, the already poor air quality is further deteriorated when the Punjab, the biggest province of Pakistan in terms of population, is surrounded by toxic smog. Each year, the smog normally occurs for 10–25 days between November and February affecting Southern and Central parts of the Punjab province and significantly decreasing visibility on roads and causing health issues. The regional data recorded in 2016–2019 about air quality names South Asian region to be at the bottom with 30 cities having worst air quality. Lahore, Pakistan's second biggest city with 11 million residents, has been recorded to experience maximum pollution. The quality of air in Lahore has dropped during the last 20 years. Its pollution concentration was 33 μg/m3 in 1998, however, by 2016, it reached 64 μg/m3—which was six times higher than the WHO standards suggesting a lost life expectancy age of 5.3 years for an average individual when comparing it to the WHO standards. The poor air quality in the third biggest Pakistani city, Faisalabad, causes the loss of an approximate 4.8 years to each individual when comparing it to the WHO standards (15). Smog, in these districts, has become a public health emergency.

**Figure 1:**
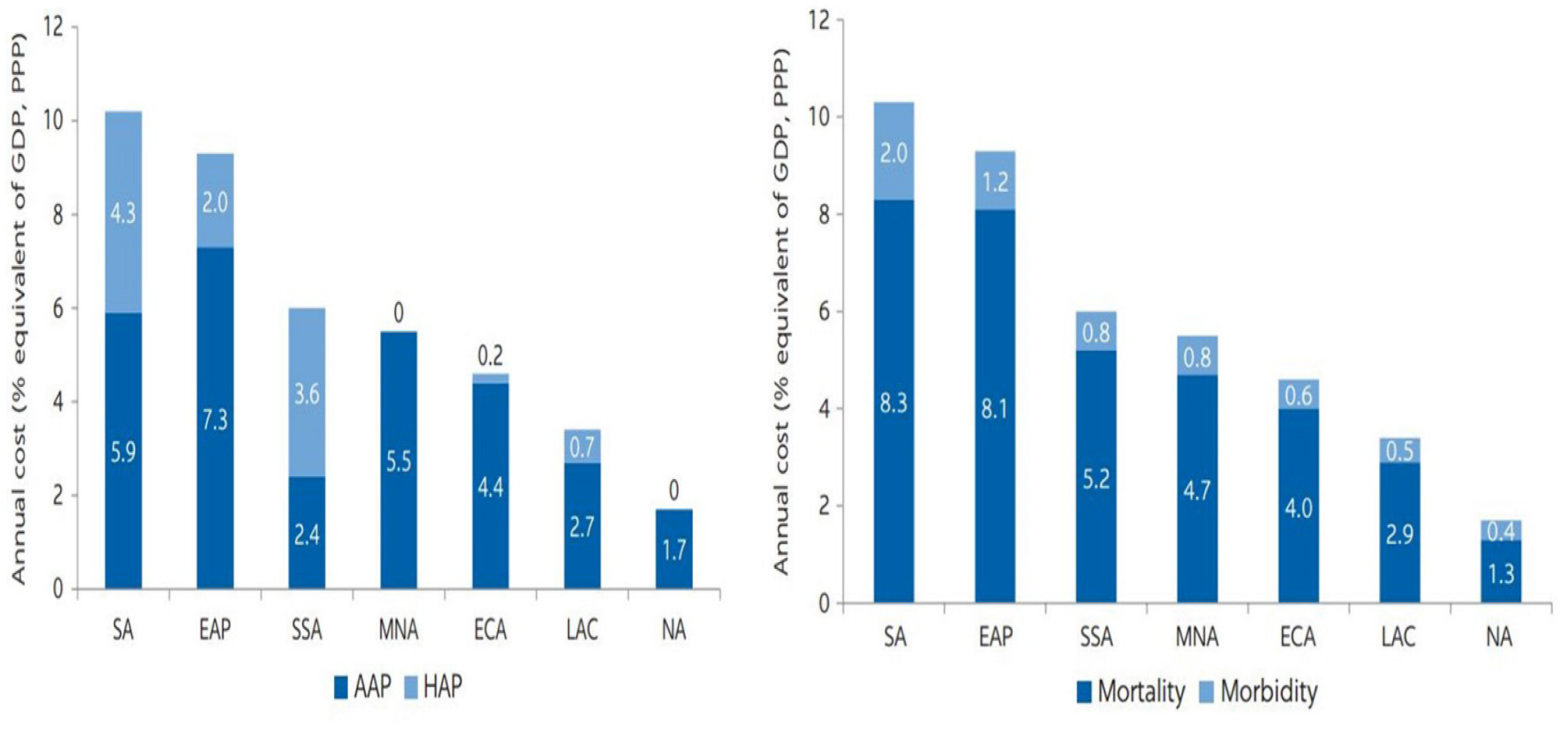
Cost of health damage from PM_2.5_ exposure in 2019 by region, % equivalent of GDP (PPP). Source: World Bank (14). EAP, East Asia and Pacific; ECA, Europe and Central Asia; LAC, Latin America and Caribbean; MNA, Middle East and North Africa, NA, North America; SA, South Asia; SSA, Sub-Saharan Africa. Numbers may not add up due to rounding.

Although the Government of Pakistan has taken many measures to address the issues relating to the health hazards caused by environmental pollution, there is still a large room for improvement. A higher ratio of GDP per capita demands effective measures for guaranteeing eco-friendly expansion. In case of weak, impractical policies, the sustainability of economic expansion may be affected (17). According to a new World Bank report (14), the global health cost of air pollution (i.e., PM_2.5_) alone is $8.1 trillion, or 6.1 percent of global GDP. In China and India, where more than half of the world's fatalities from PM2.5 air pollution occur, costs can reach 12.9 and 10.6 percent of GDP. For Pakistan this is 8.9% of GDP, even though the GDP of China is far > that of Pakistan, the health burden caused by environmental pollution in China and Pakistan is almost similar. Premature death accounts for around 85% of the entire global cost of health losses in 2019, while morbidity accounts for 15% (14). (See Figure 1).

**Figure 2:**
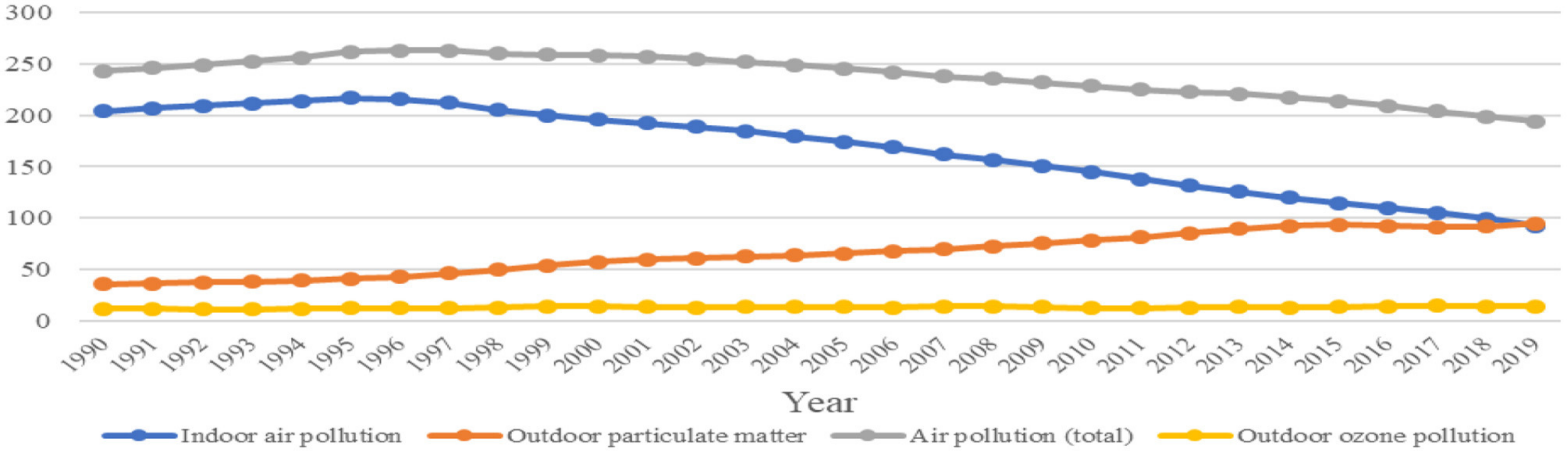
Death rates from pollution in Pakistan (18).

In the Pakistani context, air pollution makes up the biggest environmental challenge on account of defectively handled rising motorization and soaring urban industrialization to the air pollution caused by households and farmers. The automobile emissions, industrial discharge and waste, and stubble burning make up the principal sources of air pollution leading to high prevalence of respiratory diseases and premature deaths. In this respect, the statistics, supplied by the Global Burden of Disease (GBD) database, guide about the standing of Pakistan among the South Asian states and also inform about the environmental effect reduction trends in Pakistan. The recent GDB figures indicate approximately 103 deaths per 1,00,000 among Pakistani population caused by PM_2.5_ exposure in 2019 (18) (see Figure 2), which is approximately one-third < India, almost similar to Bangladeshi and Chinese contexts; however, it is 41% higher than in Indonesia, and almost double than it is in Turkey and Mexico. The statistics suggest that the states having an extensively lower health burden caused by environmental/occupational hazards possess two to three times greater the per capita GDP in Purchasing Power Parity than Pakistan. These grave consequences are supported by a World Bank (19) study that found 2.5–6.5 percent share of Pakistani GDP to be the cost of air pollution for the year 2016 (19). The improvement in air quality in Pakistan could extend the life expectancy of around 11 million people if the government can work efficiently for decreasing CO_2_ emissions (20). The World Bank has dubbed the economy of Pakistan as being “very air polluted intensive”. It is estimated that one unit of PM_2.5_ causes the wastage of 18.9 US$ of GDP per capita. Thus, air pollution in Pakistan makes up a total of 47.8 billion US$ or 5.88% of GDP as the projected economic burden. To efficiently utilize the overall natural resources, the policymakers and decision makers must consider these environmental costs to attain economic growth. This draws the attention to the pressing need to take steps efficiently in order to minimize air pollution.

The current study aims to highlight the local effects of air pollution, a domain that has drawn considerable attention in recent times. The most important reason for such extraordinary attention is rooted in the multidimensional impact of air pollution, which influences health (13), residential property value (21) and agricultural production (22). Most studies evaluating air quality and employing a life satisfaction approach have used cases of developed economies (23, 24). Silva et al. (25) has provided empirical evidence of air pollution with life satisfaction based on the data obtained from around 50 countries. Investigations carried out in the west have recognized that to prevent pollution, public sensitization and acquaintance about the environment should be increased (26, 27). Several scholars have highlighted other perspectives of environmental concerns in Pakistan. For instance, the study by Ahmed and Shafique (28) investigated the risk perception of households concerning water pollution and its consequent impacts on the health of individuals. Similarly, Khan et al. (29) investigated the long-run and contributing association between air pollution, energy use and water resources in Pakistan by employing CO_2_ emissions as a proxy for air pollution.

Their study concluded that energy use and water resources are significantly and positively associated with air pollution in both the short-run and long-run. Hussain et al. (30) discovered the adaptation and mitigation awareness regarding climate change from the general population using Pakistan's case (urban, peri-urban and rural areas).

Researchers must focus on willingness to pay assessments to understand how much better air quality matters to Pakistanis. We presently know very little about how much citizens are willing to pay for cleaner air and how this willingness to pay changes with awareness and across heterogeneous criteria such as income, education, and gender. The current study intends to first test the welfare impact of air pollution and then evaluates the WTP for the betterment of environmental quality and its determinants using the case of developing countries, such as Pakistan. In comparison to existing body of literature, the current study is different in many ways. First, the public WTP in favor of the reduction of environmental pollution and its determinants vary based on many factors: time, air pollution concentration, and yearly income rise. Hence, the current study intends to make a difference by conducting surveys in 2018 and acquires up-to-date data that bestows our results with greater practical and logical shape and artistically divides and regards both the tax payment and social contribution, thus providing the decision makers a clear public perception. Second, our study underscores the direct subjective WTP rather than indirect assessment through income that reveals a more realistic value for supporting pollution reduction at both individual and society levels. Besides the employment of common factors, the current study includes vital determinants associated with knowledge, such as risk perception and attitude for discovering the determinants of public WTP for cutting air pollution amount, thereby offering a fresh evidence for formulating explicit policies in order to deal with air pollution particularly in urban areas. Third, most of the current research on pollution control addresses micro level and policy aspect, while our research is based on microdata and offers a micro foundation for public environmental policymaking.

The rest of this paper is organized in the following way: Sections 2 and 3 supply a concise account of the literature review and key methodical approaches. Section 4 highlights the empirical results and compares them with other similar studies. The last section submits the conclusions of the study.

## Literature Review

The environmental valuation method based on life satisfaction takes into consideration indirect objective data from the respondents concerning WTP for air pollution reduction or the environment (10, 31). The sample population is asked about their socio-demographic characteristics and their life satisfaction perception. The air pollution data, deemed an external variable, is obtained from official sources. Therefore, the employment of the environmental valuation method shows the respondents' preferences and their WTP, rendering it a revealed preference method (10, 32). Since many of these techniques, revealed preferences, or stated preferences employ income variable for estimating WTP, the gains out of reduction in issues relating to environment do reveal the income level of the affected areas (33, 34).

Furthermore, employing the life satisfaction method, it is difficult to directly appraise the evaded fatalities by reducing pollution (in the damage function method, this essential value is incorporated) because it considers the perception of well-being of the survey respondents only.

Concerning the data use, two common approaches are employed in air quality valuation through the life satisfaction method (31): a macro approach (aggregate data) and a micro approach (individual data). A number of studies have employed techniques based on the employment of subjective self- reported air pollution level assessment rather than objective monitoring measures or modeling data. In this regard, Rehdanz and Maddison (35) examined the association between self-reported effect of air pollution and SWB data, supplied by the German socio- economic panel (SOEP) survey. On the same line of inquiry, Li et al. (36) carried out a study using the case of Chinese mining area. These two studies offer a careful control of demographic and socio-economic variables influencing SWB and found an inverse association between SWB and air pollution.

The models on air pollution are capable to break down the spatial data into individual level. The study by MacKerron and Mourato (37) created survey tool for collecting data on individual SWB among the Londoners. The study findings suggest that measured air pollution data negatively associate with SWB. The study by Ambrey aimed to examine multi-air pollutants in Queensland, however, out of a number of pollutants, the variable of life satisfaction was discovered to be strongly and negatively associated with PM_10_ only (38). Using the Estonian case, Orru et al. (11) obtained the individual SWB data from ESS. The data of air pollution was acquired from Eulerian air quality dispersion model with 1 x 1 km grid squares covering the entire country. The study discovered SWB to be negatively influenced by PM_10_. Based on the happiness data obtained from General Social Survey (GSS), the study by Levinson (39) found happiness and air quality to have a significant association on daily and regional levels. The study also found high levels of particulates and well-being in the USA to be inversely associated.

The study of Welsch (31) employed a macro strategy with diverse states and various air pollutants to assess the intensity of air pollution. Menz and Welsch (40) also employed macro strategy on the same footing, but they only did it for the OECD states panel data. The study by Luechinger (41) collected the data on SO_2_ concentration for about 20 years obtained from 553 German monitor stations and SWB data from SOEP survey for the same period. Correlations between the two variables were examined based on average yearly German regional data. The study controlled the variables of socio-economic characteristics and particulates and found a significant impact of SO_2_ on SWB. The study by Ferreira and Moro (42) valuated PM_10_ using Irish regional data. Based on the locations of respondents, the average yearly pollution data from the nearest monitoring stations were connected to SWB data of the respondents. The individual-level SWB was found influenced by the concentration of PM. The study of Ferreira et al. (43) primarily carried out the cross-sectional examination with spatially disaggregated data of the European region on SO_2_ to investigate individual SWB. The study found a robust inverse association of SO_2_ concentrations with self-reported life satisfaction. A latest study by Zhang et al. (40) was successful in evaluating air quality employing moment-to-moment happiness data on daily basis and local level, and discovered insignificant negative impact of bad daily air quality on overall life satisfaction, but found that poor daily air quality is likely to reduce subjective well-being and increase the chances of depression. Several authors such as Di Tella et al. (44) and Beja (45) blended microdata (life satisfaction and socio-demographic characteristics) with macro data (income and air pollution) for a number of states. These multi-regional studies offered wide-ranging interactive information between significant indicators, such as the association between air pollution and economic/environment background. Only a few studies used spatially disaggregated air pollution data at individual level.

The CV technique has recently been witnessed for its wide employment in the estimation of the economic value of non-market commodities and services, for instance environmental effect and health economics (26, 27). Clean air is considered to be a non-market commodity, which is without a market price. Therefore, this method may serve as a suitable instrument when assessing the economic worth of air pollution reduction because it can acquire market prices for non-traded commodities. In addition to the most commonly used Contingent Valuation Method (CVM), environmental researchers are increasingly utilizing self-reported “well-being or Life Satisfaction Approach (LSA)” to measure the non-marketed benefits of environmental improvements through response surveys to elicit respondents' behavior in welfare and cost-benefit analysis (46, 47). The life satisfaction method employed for estimating air quality regards the respondents' responses concerning their subjective well-being as the fundamental variable. The assessment model in this method measures experienced utility (perceived hedonic experience) instead of decision utility (associated to preferences) (48). The responses are then connected to the stated income and to the external objective air pollution data, while the demographic and other important variables are controlled. One of the main characteristics of life satisfaction method is its assumption that higher income and low environmental destruction improve the well-being of people regardless of their understanding of these determinants. Therefore, an assessment of the association between the two variables can be made (49) thereby paving way for a possibility of an indirect WTP measurement for air pollution reduction (50). The researcher then estimates the monetary value of an environmental good or service using data constituted of a set of variables. Since then, the results have been similar to those obtained using the CVM. As a result, the LSA appears as a helpful alternative to more established methodologies while also expanding the range of techniques available for environmental-economic assessment (51–55).

## The Model

The current study employs the micro-level approach. A robust life satisfaction function for evaluating micro-level data was developed by Welsch and Kühling (50) in the following shape:


(1) $$\begin{array}{l} \text{L}S_{\text{ij}}=\text{f}\left({\text{Y}}_{\text{ij}},\text{ A}{\text{P}}_{\text{j}},\;{\text{P}}_{\text{ij}},\;{\text{E}}_{\text{j}},\text{N}{\text{O}}_{\text{ij}}\right) \end{array}$$

where LS_ij_ exhibits life satisfaction level of an individual i living at j. Y_ij_ shows income level of an individual i living at j. AP_j_ displays air pollution level in physical place j. P_ij_ reveals certain noticeable characteristics of an individual i living at j. E_j_ exhibits certain other external variables living at j. NO_ij_ acts as a group of non-observables characteristics of an individual i living at j.

The data on life satisfaction (LS_ij_), personal income (Y_ij_), and the set of noticeable characteristics of an individual (P_ij_), such as age, sex, job status, academic qualification, and similar other variables, were acquired by means of a primary survey. Subjective well-being is measured as both cardinal (by psychologists) and ordinal (by economists) in the research literature (56). The results are unchanged by whether SWB is considered as cardinal or ordinal (56–58), however we apply OLS and ordered probit methods with robust standard errors to address heteroscedasticity problems (59). AP_j_ depicts the degree of air pollution (subjective measure), an external variable. The other external variables (E_j_) consider determinants absent from the survey, such as, temperature and precipitation. The set of non-observable characteristics of a person (NO_ij_) are harder to acquire and need a more comprehensive examination of personality features and other elements of well-being. The current study proposes the following econometric followed by Welsch and Kühling (52) and Frey et al. (11). The estimation aims to first test the impact of air pollution on individual happiness, represented in Eq.2. The determinants of WTP are studied, particularly the WTP are in forms of tax and personal income, corresponding to equation (3) and (4), respectively:


(2) $$\begin{array}{l} Life\;satisfaction_{i}=c+a_{1}airp_{i}+a_{2}gender_{i}+a_{3}age_{i}\;\\ +a_{4}age2_{i}+a_{5}work_{i}+a_{6}mamarital\;status_{i}\;\\ +a_{7}No.of\;child_{i}+a_{8}edu_{i}+a_{9}health_{i}+a_{10}income_{i}+\varepsilon_{i} \end{array}$$

(3) $$\begin{array}{l} Envirtax_{i}=c+a_{1}airp_{i}+a_{2}gender_{i}+a_{3}age_{i}+a_{4}age2_{i}\;\\ +a_{5}mamarital\;status_{i}+a_{6}No.of\;child_{i}+a_{7}edu_{i}+a_{8}health_{i}\;\\ +a_{9}income_{i}+a_{10}envimp_{i}+a_{12}avoidtax_{i}+a_{13}govresp_{i}\;\\ +a_{14}Lifesatisfaction_{i}+a_{15}work_{i}+\varepsilon_{i} \end{array}$$

(4) $$\begin{array}{l} Payfenvir_{i}=c+a_{1}airp_{i}+a_{2}gender_{i}+a_{3}age_{i}+a_{4}age2_{i}\\ +a_{5}mamarital\;status_{i}+a_{6}No.of\;child_{i}+a_{7}edu_{i}+a_{8}health_{i}\\ +a_{9}income_{i}+a_{10}envimp_{i}+a_{12}avoidtax_{i}+a_{13}govresp_{i}\\ +a_{14}Lifesatisfaction_{i}+a_{15}work_{i}+\varepsilon_{i} \end{array}$$

In the model, a_i_ are coefficients; *c* and ε_i_ represent the constant and the error term. The dependent variables (DV) have a discrete and ordinal form, therefore the econometric method is an Ordered Probit (OP) or an Ordered Logit (OL). Furthermore, some empirical studies that employed Ordinary Least Squares (OLS) found that this classical econometric method offers robust findings (10, 50) and there is absence of any significant variation in the impact of the relevant variables (3, 60, 61).

### The Survey Area

Two cities have been included in the current study: Lahore and Faisalabad. Lahore is the capital city of the most populated province in Pakistan which is located 31.55°N, 74.36°E. It possesses a rich culture and social diversity. It is the richest Pakistani city contributing around 58.14 billion US$ to the GDP per annum. The two biggest causes of poor air quality in Lahore are industries and vehicles, which surpass National Ambient Air Quality Standards (NAAQS). The second city under survey is Faisalabad, the third most populated city in the country, located 31°25′0″N 73°5′28″E. On account of its central location and facilitated with all types of transportations, the city is a main center for industry and distribution contributing around 20% to the provincial GDP with a yearly GDP of 20.5 billion US$. Apart from agriculture, Faisalabad is known for its agro-based and textile industries. There is no regular air quality management system in Faisalabad, and air pollution is measured on *ad hoc* basis. The current situation can worsen in the face of population growth, industrial expansion, deforestation, ever-increasing construction work, and growing motorization. Owing to its dense population, a large number of citizens of Faisalabad are at air pollution risk. The poor air quality poses serious and unavoidable effects on citizens' health. On account of the aforementioned facts and circumstances, the two cities have been selected for the assessment of household WTP for improved air quality.

#### Data Collection

Primary sources were used through a survey questionnaire in order to collect data. The questionnaire, originally designed in English, was translated into Urdu, the lingua franca of the area, so that accurate and true responses could be obtained. Before administering the study questionnaire, a pilot survey was carried out on November 5, 2017 to verify if the questionnaire items covered everything in accordance with the study objectives. The pilot study included academics and researchers numbering 50, who were satisfied with the questionnaire. The study survey was carried out between December 2017 and February 2018 employing face to face survey of 600 households which were selected randomly. During the process, each question was explained to the respondents in their native language for complete understanding and for obtaining true responses. Most respondents consumed 30–40 min in completing the questionnaire. The total of 700 questionnaire forms (350 from each city) were filled and obtained, but 100 were rejected on account of incomplete filling, mistaken filling, forged filling and so forth, making the total sample size to 600.

### Variable Description and Measurement Method

####  Dependent Variables

The self-reported “well-being or Life Satisfaction Approach (LSA),” in addition to the most commonly used Contingent Valuation Method (CVM), is gaining popularity among environmental researchers for measuring the non-marketed benefits of environmental improvements through the use of response survey to elicit respondents' behavior in welfare and cost-benefit analysis (46, 47). By developing an econometric model, this technique is frequently employed in numerous aspects of environmental valuation, minimizing the potential difficulty with the contingent valuation method of having skewed data from respondents during survey. Because individuals are asked to score their overall happiness level, the research considers data on life satisfaction or SWB acquired using self-reported questionnaires to be the best acceptable measure for persons' utility. The researcher then quantifies the monetary value of an environmental good or service using data constituted of a set of factors. Since then, the results are identical to those obtained using the CVM. As a result, the LSA emerges as a helpful complement to more established methodologies, while also broadening the range of tools accessible for environmental economic valuation (51–53). We adopted unipolar liket scale question to measure the self-reported SWB from respondents; “How satisfied are you with your life as a whole?” in the light of extant literature (62, 63). The responses were recorded on the 0 to 10 scale, where 0 indicated “very dissatisfied” and 10 “very satisfied” (64, 65). Supplementary Table S1 illustrates the distribution of responses to the life satisfaction perception for urban areas in Punjab. A high percentage of households reveal low level of life satisfaction: 45.03% of individuals with life satisfaction obtained scores between 7 and 10.

The survey contains two questions about the WTP for environmental quality improvement. First: “*I would agree to an increase in taxes if the extra money were used to prevent environmental pollution” and* translates to the variable envtax. The value 1 to 4 is awarded to responses of *strongly disagree, disagree, agree and strongly agree*, with greater values linked to higher willingness of tax payment. Second: “*Are you willing to sacrifice part of your income to lower the risk of extreme events, such as heat waves, smog pollution, air quality, floods, droughts and hurricanes, which occur because of climate change?”* in accordance with the variable *income contribution* with values 1, 2, 3, 4 and 5 representing:

Yes, I am willing to sacrifice between 0.1 and 1% of my income

Yes, I am willing to sacrifice between 1 and 5% of my income

Yes, I am willing to sacrifice between 5 and 10% of my income

Yes, I am willing to sacrifice more than 10% of my income

No.

Different from the mandatory tax payment imposed by governments, the personal payment reveals more like a social contribution. The two variables show varied payment preferences for pollution control, with mean 2.31 and 2.21, respectively. In addition, 65.34% of the sample population opts to agree or strongly agree, whereas 47.17% respondents choose to give up 1% to 5% of their personal income. 13.50% refused to share any amount of their income. The common people are found to have comparatively high WTP. Moreover, comparing mandatory and voluntary cost bearing for the prevention of pollution, the tax system appears less favorable than that of personal payment.

#### Independent Variables

In the current study, the air pollution index, used as a main variable concerning its impact on happiness and WTP, covers two aspects of measurement: objective pollution record, which is mostly represented through the density of SO_2_, NO_2_, PM_10_, and PM_2.5_, and subjective pollution, which is the respondent's perception. The objective measurement reveals only the absolute value of pollution level, whereas the subjective assessment contains both the objective air pollution and the relative level of satisfaction comparing the air quality in other parts of the region. Because of its recording mostly at municipal and occasionally the district level, the objective pollution level density may vary significantly even within a perimeter of a single city. Therefore, the current study uses subjective perception about local air pollution, which appears to be wide-ranging, powerful, and apparent to individuals (37). Seriousness of air pollution is measured using four-point Likert scale from “Not serious at all” = 1 to “Very serious” = 4 but for regression purpose we classified this index to *not serious at all and very serious*, and is rated as 1 and 2. 87.5% of the people of Punjab are not aware of the air pollution problem to which they are exposed day after day. 12.5% rates the intensity of air pollution as very serious. The results show that only 13% of respondents are not willing to pay for income contribution for environmental quality improvement reporting indifferent attitude and insufficient knowledge of the environment.

The survey contains the main study variables about environment or tax attitude. Taking care of the local environment is important and is rated as 1 in envimp variable, otherwise, 2. The envimp evaluates people's concern on the environment. Another variable govtresp characterizes the responsibility of environmental organizations, corresponding to the reply of the government must decrease environmental pollution; however, this ought not to put any financial burden on me. The values range from 1 to 4, with various degrees of agreement and disagreement responses. People's attitude toward environmental organizations and government play an important role in people's WTP for the prevention of pollution, and it also points to trust in the markets. The trust in environmental organizations and trust in government are indicated by envot and trustgov, with various degrees ranging from 1 to 4. Finally, people's attitude toward tax compliance is a major contributing factor for their inclination toward tax payment to protect the environment. The study uses avoid tax to measure incentives on tax evasion, with integer value “Always be justified” = 1 to “I do not know” = 4. These variables reveal individual attitude, social trust, and sense of duty influencing the WTP in return.

The estimation also includes a number of socio-demographic variables: gender, age, academic qualification, job status, marital status, and having a child. The education variable entails the number of years in formal education: 0, 5, 8, 12, 14 and 16 equivalent to no formal education, primary school, secondary school, high school, university, master's and above. About the health status of the respondents, the question is: “*Have you suffered severe health problems over the past 2 years?”*. The study reveals that the maximum number of the respondents (32.76%) has a monthly income of PKR17, 001 to PKR22, 000. The second-largest category belongs to the people (26.33%) possessing the monthly income of PKR22001 to PKR27, 000, whereas 16.17% and 9.5% of the respondents earn monthly PKR12, 001-PKR17, 000, and PKR7, 000 - PKR12, 000, respectively. Only 9% of respondents have a monthly income of more than PKR27, 000.

## The Regression Results and Discussions

### Results

The purpose of employing ordered-probit models is to approximate the probability of respondents. Table 1 displays the regression results from model (1). In model 1, the regression results reveal that the coefficient of air pollution is negative, −2.967. There is a significantly positive relationship between life satisfaction and the annual household income. Furthermore, results suggest that age and health exert significantly negative impact on SWB. It implies that if the respondent is elder with health issues, he or she is less likely to be happy (40). The sign, coefficient and significance of both OLS and Probit estimation, is consistent. As for other variables, happiness signifies a U-shape association with age (62). In line with general perception and existing literature, education and income positively influence personal happiness. The variable gender turns out to be insignificant, which aligns with (66, 67), that there is no significant variation in happiness perceptions of males and females. Air pollution aggravates personal happiness as proven; therefore improvement in the quality of the environment improves personal and social welfare. The protection of the environment needs financing and the following regressions estimate the determinants that affect the WTP for pollution reduction.

**Table 1:** The effect of air pollution on subjective life satisfaction.

	**Dependent variable: life satisfaction**	
**Variables**	**OLS**	**Ordered–probit**
Air pollution	−2.967^***^	−1.492^***^
	(0.270)	(0.139)
Gender	0.096	0.026
	(0.179)	(0.085)
Age	−0.080^*^	−0.026^**^
	(0.178)	(0.336)
Age^2^	0.474^*^	0.042^*^
	(0.178)	(0.084)
Edu	0.333^***^	0.146^***^
	(0.068)	(0.033)
Work	−0.033	−0.016
	(0.052)	(0.025)
Marital status	0.111^*^	0.067^**^
	(0.081)	(0.044)
Children	0.134^*^	0.059^**^
	(0.069)	(0.033)
Income	0.208^***^	0.113^***^
	(0.078)	(0.037)
Health issues	−0.417^**^	−0.231^*^
	(0.250)	(0.120)
Observations	600	600
R^2^ Pseudo R^2^	0.581	0.19

*Standard errors in parentheses*.

*** *p < 0.01*,

** *p < 0.05*,

* *p < 0.1*.

Model 2 results are highlighted in Table 2. New DV variable WTP for environmental tax is introduced in this model. The air pollution coefficient is significantly negative (68). This result shows that the individual perception of air pollution does not reveal significance on people's willingness to pay, implying that those who think air pollution is dangerous are most likely not to pay more tax to reduce pollution.

**Table 2:** Determinants of the tax payment for pollution prevention.

	**Dependent variable: envtax**	
**Variables**	**OLS**	**Ordered–probit**
Life satisfaction	−0.020^*^	−0.029^**^
	(0.014)	(0.021)
Air pollution	−0.026^**^	−0.039^**^
	(0.013)	(0.051)
Gender	0.014	0.021
	(0.062)	(0.091)
Age	−0.114	−0.172
	(0.249)	(0.363)
Age^2^	0.015	0.025
	(0.062)	(0.091)
Edu	−0.059^**^	−0.087^**^
	(0.024)	(0.036)
Work	−0.016	−0.023
	(0.018)	(0.027)
Marital status	0.027^**^	0.039^**^
	(0.032)	(0.047)
Children	−0.059^**^	−0.085^**^
	(0.024)	(0.035)
Income	0.016^**^	0.021^*^
	(0.027)	(0.041)
Health issue	0.003	0.008
	(0.088)	(0.127)
Env Priority	0.016^*^	0.022^*^
	(0.059)	(0.087)
Env org trust	0.019^**^	0.028^**^
	(0.035)	(0.051)
Avoid tax	−0.084^***^	−0.125^**^
	(0.036)	(0.053)
Govt reduce pollution	−0.170^***^	−0.249^**^
	(0.035)	(0.052)
Observations	600	600
R^2^/ Pseudo R^2^	0.395	0.045

*Standard errors in parentheses*.

*** *p < 0.01*,

** *p < 0.05*,

* *p < 0.1*.

The coefficient of environment priority and environment organization trust are significantly positive. It reveals that the likelihood of positive WTP carries a positive association with public's trust in the environmental organization and priorities for environment, which implies an increasing trust in the environmental organization and environmental priority, which implies that an increasing trust in the environmental organization contributes to a higher probability of having a positive WTP (69).

Model 3 results in Table 3 highlight the factors affecting WTP in relation to individual income, referred to as social contribution, different from the mandatory tax payment. We run the regression with OLS and Ordered probit models, where the results are not much different. The study finds that air pollution is positively significant at 5% level with income contribution, implying that those who think air pollution is dangerous are most likely to pay more social payment to reduce pollution. The awareness about environmental protection is more associated with WTP than awareness about air pollution's effect on health and quality of life (68–70). The results show that almost 87.5% of the inhabitants of Faisalabad and Lahore do not consider the air pollution problem a serious problem to which they are exposed day after day. The lack of awareness about the severity of air pollution in Pakistan may cause to reinterpret the people's WTP more taxes for clean air. Therefore, educating the general population about pollution prevention should be a significant step toward environmental governance.

**Table 3:** Determinants of the social payment for pollution prevention.

	**Dependent variable: income contribution (Payfenvir)**	
**Variables**	**OLS**	**Order–probit**
Life satisfaction	0.015^**^	0.039^*^
	(0.023)	(0.021)
Air pollution	0.021^**^	0.024^**^
	(0.074)	(0.071)
Gender	−0.011	−0.001
	(0.098)	(0.091)
Age	−0.328^**^	−0.319^*^
	(0.391)	(0.363)
Age^2^	0.085	0.082
	(0.098)	(0.091)
Edu	0.006	0.028
	(0.038)	(0.035)
Work	0.083^***^	0.073^***^
	(0.029)	(0.027)
Marital status	−0.105^**^	−0.099^**^
	(0.050)	(0.047)
Children	−0.048	−0.052
	(0.038)	(0.035)
Income	0.081^*^	0.072^*^
	(0.043)	(0.040)
Health issues	0.057	0.078
	(0.137)	(0.128)
Env Priority	0.051^**^	0.069^**^
	(0.093)	(0.087)
Env org trust	0.099^*^	0.093^*^
	(0.055)	(0.051)
Avoid tax	−0.041^***^	−0.067^**^
	(0.056)	(0.052)
Govt reduce pollution	−0.416^***^	−0.306^***^
	(0.055)	(0.053)
Observations	600	600
R^2^ / Pseudo R^2^	0.132	0.040

*Standard errors in parentheses*.

*** *p < 0.01*,

** *p < 0.05*,

* *p < 0.1*.

The social WTP must not be underestimated because it implies if individuals trust environmental organizations for the protection of environment (envot). Moreover, it also involves individual perception about pollution reduction being a social duty rather than a governmental responsibility (govt resp), higher incentives of tax payment (avoid tax), and family income (income), and they all persuade citizens to pay more for environmental protection. The environmental organizations, excluding the governmental agencies, serving as main bodies aiming for improvement in environmental quality are likely to have a significant impact on WTP (at 1% level) depending on using the donations capably and carrying out adequate environmental work.

### Further Discussion

The results suggest that Pakistanis are not aware of air pollution's effect on health and quality of life. They are willing to pay more in social payment in return for different environmental attributes (such as climate change mitigation, water pollution control, and control of air pollution). However, the variable, importance of environment, appears significant at 1% level signifying that those who care more about the environment are most likely to pay more in tax in return for a better environment. The range of people's willingness to consent to a tax increase for pollution prevention depends not on the severity of air pollution but on their perception of damage to the environment and their preference for cleaner air (26, 27, 70). Moreover, the judgment on one's responsibility of environmental protection (govt resp) negatively affects WTP, implying that individuals may likely resist an increase in tax if they think pollution reduction is a governmental obligation rather than a social one. Citizens' trust in government plays a pivotal role in people's WTP in taxes for pollution reduction (27). Public service also includes the improvement of environmental quality, which is a common choice, and tax payment is used as the most convenient method to look after environmental management. In this connection, people's trust in the government being capable to decrease pollution plays a central part in people's perception of their contribution to tax for environmental improvement. This clearly suggests that trust-building by the government and the consequent people's trust in government serve as the foundation of tax collection and implementation of pollution prevention plan. The variable avoid tax about tax payment, symbolizing the individual behavior of general tax evasion and deficient social responsibility, is found to have a negative association with WTP tax for the improvement of the environment. Another determinant that is likely to affect WTP is family income, which reveals that families with higher financial status are likely to be comparatively less sensitive and more demanding about the improved environment (68). Factors influencing the WTP also include the household income, where people with higher income are relatively less sensitive in money and more desirous of a pleasant environment, thereby more willing to pay for pollution reduction.

In most developing countries including Pakistan, the most significant causes for indifference toward environmental concerns is people's low level of sensitization. This apathy and lack of understanding, coupled by uncompromising nature act as fundamental determinants for pollution and the consequent effects on health. With reference to Pakistan, the major decision-makers ought to be taken on board concerning the measures they have taken to espouse the policy recommendations forwarded for the resolution of issues relating to air pollution. Due to Pakistan's lack of political stability throughout its political life, several most pressing issues have failed to seek governmental attention, particularly environment and health. The Pakistani policy feedback mechanism at regulatory authorities is absent. The unsuccessful legislation is caused by insufficient support from the policymakers and low awareness among the ordinary population as well as members of parliament. With reference to Pakistan, efforts should be made for the formulation of well-defined institutional roles and responsibilities and their successful coordination. In order to fulfill adaptation and mitigation aims in all sectors, Pakistan needs to introduce modern technology and technological partnerships. In order to solve the repeated damage caused by extreme weather events and air pollution, Pakistan undertook a Technology Need Assessment (TNA) in 2017. The evaluation might lead to Pakistan developing a comprehensive plan for national climate change mitigation technology development. Punjab province has long battled to decrease pollution. Punjab local government should take the following steps to control air pollution; Bans on municipal garbage burning, adoption of the Punjab Clean Air Action Plan, public release of air quality data, and the implementation of digital smog response systems. The negative relationship between pollution and health is well documented in the literature, yet results from specific regions are frequently inapplicable to other situations. Pakistan has to do a better job of measuring the entire costs of long-term air pollution exposure. The research must not just look at mortality, morbidity, and cognition, but also at the effects of pollution on behavioral decisions including fertility, migration, time usage, and defensive spending. Such proof of effect will highlight the potential consequences of air pollution and encourage governments to take action. Researchers must focus on willingness to pay indicators to understand how much improving air quality matters to Pakistanis. We presently have limited evidence on how much individuals are ready to pay for better air quality and how this desire to pay varies with information and across heterogeneous criteria such as income, education, and gender. Pakistan may benefit from the experiences of other developing countries in regulating air quality. Despite the fact that many Indian cities suffer from severe pollution, many provinces have begun to implement evidence-based policies to enhance air quality. A decade ago, China had some of the world's worst air quality, but it has since made significant progress in lowering pollution. Some of the policies implemented by both countries can be used to improve Pakistan's pollution policy.

## Conclusion

Out of a host of challenges facing Pakistan, the environmental problem has become a hot and daunting issue. Reduction in air pollution and protection of the environment demands a huge amount of money that the citizens must pay in direct or indirect ways. Thus, investigating the effect of air pollution on individual welfare, the people's WTP for pollution prevention, and socio-economic factors influencing WTP deserves immense worth in environmental policymaking. The current study evaluates the problem through a micro-level prism employing primary survey data obtained from two major Pakistani cities: Lahore and Faisalabad and finds that personal happiness is significantly affected by air pollution. Two aspects of WTP, obligatory tax payment and voluntary social contribution, are used. The social WTP is found to be a little > the WTP obligatory tax, proposing a governmental preference of voluntary contribution to a tax increase for meeting the expenditure on the environment. Furthermore, the determinants affecting the WTP are similar: Household income, trust in government or the environmental organization, incentives for tax payment, duties on environmental protection. It is not the gravity of air pollution that generates an impact on WTP rather it is the people's concern for the environment that influences WTP.

The current investigation offers the following policy recommendations to Pakistani policymakers to successfully combat the pressing problem of air pollution. Concerning the challenges posed by air pollution and its subsequent effects on the environment and health, Pakistani authorities should address the lack of sensitization by raising awareness among all the stakeholders. The stern legislative instrument should be used to reduce air pollution. Subsidies and incentives should be introduced in favor of eco-friendly manufacturing. Extensive monitoring of air pollutants should be ensured. Improved air pollution management practices should be adopted. Emission inventories and source apportionment of pollutants should be developed in order to strategize affordable and effective air pollution control plans. The governmental policy implications to get public support and execute environmental policies successfully may include tax collection and utilization founded on transparency and pragmatism, public awareness of environmental protection, and improved social responsibility. Progressive taxation should be devised since people with the sound financial condition are able and more likely to pay taxes. Moreover, pollution reduction cannot be the sole responsibility of the government. Since the public pays the amount allotted for the protection of the environment, the government ought to generate consensus among the general population about environmental importance, individual responsibility, and social duties, thereby lessening the free-rider problem and increasing the WTP.

## Data Availability Statement

The raw data supporting the conclusions of this article will be made available by the authors, without undue reservation.

## Author Contributions

MR: introduction, conceptualization, methodology, and data collection. JS: literature review and reviewing. AL: results, discussion, and reviewing. YL: concluding remarks and implications. All authors contributed to the article and approved the submitted version.

## Funding

This research was supported by the China Postdoctoral Science Foundation (Grant Number: 2021M700261). MR would like to thank International Postdoctoral Exchange Program, Shandong University, under the China International Postdoctoral Exchange Fellowship Program for financial support.

## Conflict of Interest

The authors declare that the research was conducted in the absence of any commercial or financial relationships that could be construed as a potential conflict of interest.

## Publisher's Note

All claims expressed in this article are solely those of the authors and do not necessarily represent those of their affiliated organizations, or those of the publisher, the editors and the reviewers. Any product that may be evaluated in this article, or claim that may be made by its manufacturer, is not guaranteed or endorsed by the publisher.
