# Supplementary material for: Assessment of Willingness to Pay for Pollution Prevention, Health and Happiness: A Case Study of Punjab, Pakistan
Source: Front Public Health. 2022 Jun 20;10:825387. doi: 10.3389/fpubh.2022.825387 (PMC9251188; doi:10.3389/fpubh.2022.825387)
Supplement: Supplementary file 1 [file Data_Sheet_1.pdf]

Appendix

Table 1. Variables' details

Variables	Details	Type
Gender	Male and female	1 = Female<br>2 = Male
Age	Participants' age	1 = 18–25 years<br>2 = 26–43 years<br>3 = 44–61 years<br>4 = 62 and above years
Education	Qualifications' background	1= "5Years"School (Primary)<br>2= "8 Years" School (Middle)<br>3=Secondary General<br>4= Intermediate<br>5=Graduate<br>6=Postgraduate<br>7=Higher Education<br>8=Other Please Specify
Income	Monthly income of households	1 = Less than PKR7000<br>2 = PKR7000–PKR12000<br>3 = PKR12001–PKR17000<br>4 = PKR17,001–PKR22,000<br>5 = PKR22,001–PKR27000<br>6 = Above PKR27000
Work	Employment status	1=Employed<br>2=Personal Business<br>3=Unemployed<br>4=Part time<br>5=Retired<br>6=Student<br>7=Housewife<br>8=Others
Marital Status	Household marital Status	1=Couple<br>2=Widow/Widower<br>3=Divorced<br>4=Single
Children	Number of Children	1=None<br>2=1 Child<br>3=2 Children<br>4=3 Children<br>5=4 Children<br>6=5 or more Children
Life Satisfaction	Satisfaction with life in general	Measured using ten-point Likert scale from "Completely dissatisfied" = 1 to "Completely satisfied" = 10

Health Problem	Have you suffered severe health problem over the past 2 years?	1=N0<br>2=Yes
Environment Importance	Environment Protection VS Economic Growth & Creates more Job	1= Environment Protection<br>2= Economic Growth<br>3=Do not Know
Air Pollution	Seriousness of Air pollution	Measured using four-point Likert scale from “Not serious at all” = 1 to “Very serious” = 4
Environment organization trust	How much confidence you have in Environmental organizations?	Measured using four-point Likert scale from “Strongly agree” = 1 to “Strongly disagree” = 4
Income Contribution	Social Payment to reduce Air Pollution	Measured using five-point Likert scale from WTP income part between “0.1% and 1%” = 1 to “more than 10%” = 4, Do not want=5
Environmental Tax	Tax use to prevent environmental pollution	Measured using four-point Likert scale from “Strongly agree” = 1 to “Strongly disagree” = 4
Government Responsibility	Govt should control air pollution, but it should not cost me	Measured using four-point Likert scale from “Strongly agree” = 1 to “Strongly disagree” = 4
Avoid tax	Cheating on taxes	Measured using four-point Likert scale from “Always be justified” = 1 to “I do not know” = 4

Table 2. Descriptive Statistics.

Variables	N	Mean	Sd	Min	Max
Gender	600	1.562	0.497	1	2
Age	600	1.912	0.706	1	3
Work	600	1.900	1.728	1	8
Education	600	4.48	1.53	1	8
Marital status	600	1.582	1.045	1	4
No. of Children	600	2.618	1.410	1	6
Income	600	3.902	1.315	1	6
Life satisfaction	600	5.735	2.523	1	10
Health issue	600	1.853	0.354	1	2
Env Priority	600	1.495	0.520	1	3
Air pollution	600	1.125	.330	1	2
Envir org trust	600	3.178	0.888	1	4
Income contribution	600	2.317	1.243	1	5
Envir tax	600	2.218	0.776	1	4
Govt reduce Pollution	600	2.395	0.887	1	4
Avoid tax	600	1.542	0.830	1	4

Table 3. Pair wise correlation.

Variable	1	2	3	4	5	6	7	8
1.Life satisfaction	1							
2.Air pollution	-0.44*	1						
3.Income	0.23**	-0.07*	1					
4.Health issue	-0.04**	0.01*	0.08*	1				
5.Payfenvir	-0.03***	0.05*	-0.09*	-0.01	1			
6.Envirtax	-0.10*	0.07***	-0.05**	-0.02	0.17***	1		
7.Govtresp	0.01*	-0.04*	0.05	0.04	-0.03***	-0.21***	1	
8.Avoid tax	0.05**	-0.06**	-0.02	-0.07	0.03	0.09*	0.01	1
